# Practical Text-Book of Midwifery for Nurses and Students

**Published:** 1900-12

**Authors:** 


**Practical Text-Book of Midwifery for Nurses and Students.** By ROBERT JARDINE, M.D. Pp. xv., 245. Edinburgh: William F. Clay. 1899.

This little book has certain merits in itself, in that it contains many sound and useful axioms of midwifery practice.

As a text-book for nurses it has the fault of being over-burdened with detail of processes, a knowledge of which is not necessary to a nurse or midwife, while in many important details it lacks precision.

For example, we cannot understand the necessity for burdening a book for nurses with descriptions and figures of pathological curiosities such as the forms of contracted pelvis described by Robert and Naegele, with which few medical men are acquainted except as depicted in plates or in the form of plaster-casts of the originals.

We cannot commend the author in his advice as to the administration of morphia in the shape of ¼-grain suppository, or of laudanum in doses of 10 to 15 minims, by the nurse. This, in our opinion, should not be done except under medical advice.

Under the treatment of *post partum* relaxation the nurse is advised to give "ergot every four hours;" no dose is mentioned.

"Sapræmia" is to be treated by the administration of a vaginal douche: if the temperature remains high medical or rather skilled assistance is to be sought, thus allowing plenty of time for general infection to take place; but "septicæmia" demands medical assistance.

No nurse or midwife should be taught that she is to treat such cases on her own responsibility, or that she is to wait for certain symptoms before sending for medical assistance.

In describing the preparation of artificial human milk, the author advises the adding of the whey produced by the addition of rennet to part of the milk, and extraction of the curd, to the remainder of the milk without any furthur treatment. The result if the whey is not boiled first is to cause the whole of the milk to curdle and become useless.

We notice several clerical and typographical errors: "a pulse of *120°* (degrees)," "decidu-*æ*," "a man *by the name of*," are a few of these.

